# Utilization of Geotextile Tube for Sandy and Muddy Coastal Management: A Review

**DOI:** 10.1155/2014/494020

**Published:** 2014-05-13

**Authors:** Siew Cheng Lee, Roslan Hashim, Shervin Motamedi, Ki-Il Song

**Affiliations:** ^1^Department of Civil Engineering, Faculty of Engineering, University of Malaya, 50603 Kuala Lumpur, Malaysia; ^2^Department of Civil Engineering, Inha University, 100 Inha-ro, Nam-gu, Incheon 402-751, Republic of Korea

## Abstract

Threats to beaches have accelerated the coastal destruction. In recent decades, geotextile tubes were used around the world to prevent coastal erosion, to encourage beach nourishment, and to assist mangrove rehabilitation. However, the applications of geotextile tube in sandy and muddy coasts have different concerns as the geological settings are different. Applications of geotextile tubes in sandy beaches were mainly to prevent coastline from further erosion and to nourish the beach. However, for the muddy coasts, mangrove rehabilitation and conservation were additional concerns in coastal management schemes. The mangrove forests are natural barriers which can be found on the muddy coasts of many tropical countries. In this paper, the viability of geotextile tubes in sandy and muddy beaches was analysed. The advantages and disadvantages of the utilization of geotextile tubes in coastal management were discussed based on the experiences from the tropical countries such as Mexico, Malaysia, and Thailand. From the case studies, impressive improvements in coastal restoration after installation of geotextile tubes were shown. Based on the discussion, several recommendations to improve the application of geotextile tubes were suggested in this paper.

## 1. Introduction

Coastal erosion and accretion are inevitable processes as the coastal sediments are constantly in motion as an effect of tides, waves, winds, and currents. Human activities such as sand dredging and harbour construction have been disrupting the continuity of sediment transport and accelerate the coastline erosion. In addition, climate change, sea level rise, and storm surges added another layer of complexity to the eroding coasts [1]. Coastal structures are built to prevent further erosion of shorelines as well as restoring the eroded beaches to their initial phase. Hence, without coastal protection measures, eroded coastline can ravage the public properties. Therefore, coastal structures are imperative to protect the environment, ecology, infrastructures, and economic activities near shore [2].

The conventional coastal structures (i.e., breakwater, groins, revetment, and seawalls) have been constructed using wood, rock, and concrete [3–6]. Nonetheless, the recent consideration of environmental approaches and the limited resources of natural rocks in certain regions led to an increase in the application of geosynthetics in coastal protection [7, 8]. Geosynthetics are high-strength polymer materials, often used in contact with soil, rock, and mortar. There are six main types of geosynthetics which include the geotextile, geogrid, geonet, geomembrane, geosynthetic clay liner, and geocomposite [9]. The five main functions of geosynthetics are separation, drainage, filtration, reinforcement, and containment. Therefore, for the past three decades, geosynthetics have been used in the construction of coastal structures to maintain the dynamic equilibrium of coastline [10–14].

Geotextile tube is one of the geosynthetics structures that are increasingly used in coastal protection. Geotextile tubes are made from high-strength geosynthetic fabrics that allow the water to flow through pores while retaining the filling materials. They are widely used for dewatering, flood control, and coastal protection. Geotextile tube can be used in various conditions as a result of the low consumption of construction cost and time, requirement of simple equipment, and low-skilled workers [15–17]. Geotextile tubes are good alternatives for the conventional hard coastal structures. For example, Heibaum [18], Álvarez et al. [14], Saathoff et al. [19], and Pilarczyk [20] described the successful application of geotextile tubes as armouring layers, filtering layers, and scour protection for different types of coastal structures. However, they have lower resistance to wave attacks. In addition, there is insufficiency in design code for geotextile tube application.

Sandy beaches and mudflats are two types of geotechnical stratum of the coasts. The morphological characteristics of sandy coasts and mudflats vary due to the variation in wave propagation towards the beach. For example, sandy beaches are continuously reshaped by higher intensity waves and tides. Fine sand will be washed away and leave coarse sand to withstand the wave forces. On the other hand, mudflats that are composed of sand, clay, or fine silt occur at the coastlines which are protected from strong waves. Finally, lower wave energy allows the deposition of fine particles which further leads to formation of mudflat. In many tropical countries, mangroves grow along the muddy coast. Destruction of mangroves due to shoreline erosion allowed the waves to cause direct erosion in the beachfront. Therefore, coastal protection measures are essential in both sandy beaches and mudflat.

The conduction of the coastal protection measures is significantly site dependent as there are various interrelated and locally diverse parameters. These parameters include sediment properties, water elevation, wave and current characteristics, geomorphology setting, protection goals, required intervention level, safety level, and social, economic, and politic factors [21, 22]. Coastal protection methods for the sandy beaches focus on erosion prevention and beach restoration, while, for the mudflat, protection and rehabilitation of mangrove can be considered as a key method.

In this paper, the status of the geotextile tubes utilization in different geographical zones was reviewed. Further, the differences in design considerations, ability for sediment accumulation, and mangrove rehabilitation between sandy and muddy coast were discussed. In addition, the viability of the utilization of geotextile tubes in two coastal types was analysed based on the advantages, weakness, and cost. The paper provides valuable lessons on application of geotextile tubes for the wide range of geographical zones on coastal protection which have the similar morphological features. Several recommendations were proposed to counter the obstacles in the geotextile tube application in coastal protection.

## 2. Application of Geotextile Tubes in Sandy Coasts

Applications of geotextile tubes in sandy coasts with the main aim of protecting the shoreline from further erosion were presented through the case studies of Yucatan's coast in Mexico and Teluk Kalong's coast in Malaysia. The utilization objectives, method, design considerations, and lessons learned from the case studies were introduced.

### 2.1. Case I: Sandy Coast of Yucatan, Mexico

**Figure 1:**
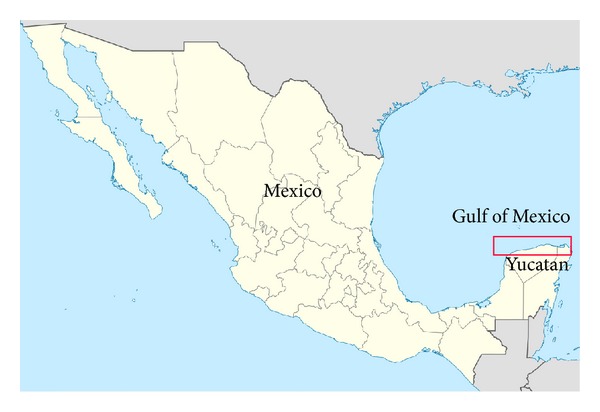
Eroded area at the northern coast of Yucatan, Mexico.

Northern coast of Yucatan, Mexico has been experiencing severe erosion since the last three decades. Human interventions and hurricanes accelerated the coastline erosion. In 2002, many areas along the coast had completely eroded. Therefore, coastline stabilization was urgently needed to prevent devastation on the shoreline and public properties. The red box in Figure 1 shows the eroded area at Yucatan, Mexico.

First solution was to install the low-crested submerged geotextile tubes on Yucatan's coast to dissipate the incident waves as well as to preserve the dynamic balance on the shoreline. Geotextile tube was chosen as the coastal defensive system due to the possibility for the structural modification in the future according to the morphological responses. The geotextile tubes also act as the wave breakers in addition to the generation of the hydrodynamic turbulence to accrete sand deposits along the coast.

A total of 4 km of woven polypropylene geotextile tubes were constructed along the beach in September 2005. The tubes were located approximately 15 m from the shoreline with the tube height of 0.9 m. Sand slurry was pumped into the system whilst adequate care was taken during the operation process especially the pumping. Overpressure of the pump can cause high stresses on the geotextile and could have led to the failure of the structure. Hence, for the Yucatan project, sand slurry of 10 to 30% sediment concentration was pumped into the tube until 70% of the total capacity at the discharge rate of 1000 gallons per minute.

**Figure 2:**
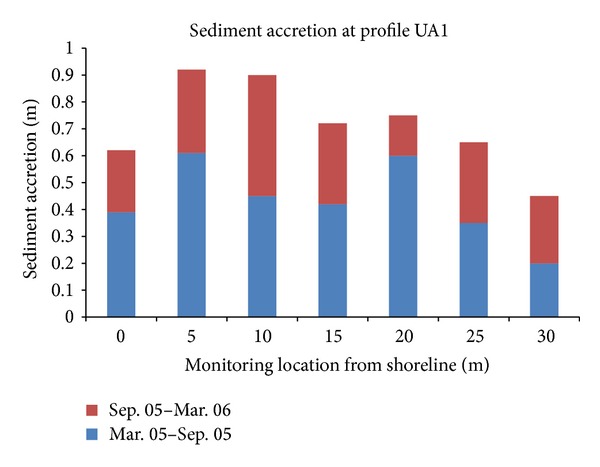
Sediment accretion at profile UA1, Project Yucatan, Mexico (adapted from Álvarez et al., 2006).

Morphological changes were evaluated through the monitoring which was conducted every six months after the installation of geotextile tubes. Álvarez et al. [14] reported sediment accretion ranging from 0.45 to 0.90 m at different monitoring locations.  Figure 2 shows the sediment accretion at one of the monitored profiles in the project. Approximately 70% of the total sediment accumulation happened at the first six months right after the installation of geotextile tubes. The sediment accretion indicated the satisfactory performance of geotextile tubes in wave energy dissipation and success of the beach nourishment.

Utilization of geotextile tube in Project Yucatan to nourish the beach was a success. Detailed monitoring results can be found in [14]. However, two issues were raised for future concerns in this project, the ultraviolet (UV) resistance and the seam strength of geotextile tube. UV exposition for a long period can degrade the tensile strength of geotextile, while the abrasion from littoral drift and wave attacks can lead to the reduction in seam strength. These two concerns can cause the loss of mechanical properties of geotextile tube in the long term and are unpredictable in design.

### 2.2. Case II: Sandy Coast of Teluk Kalong, Malaysia

**Figure 3:**
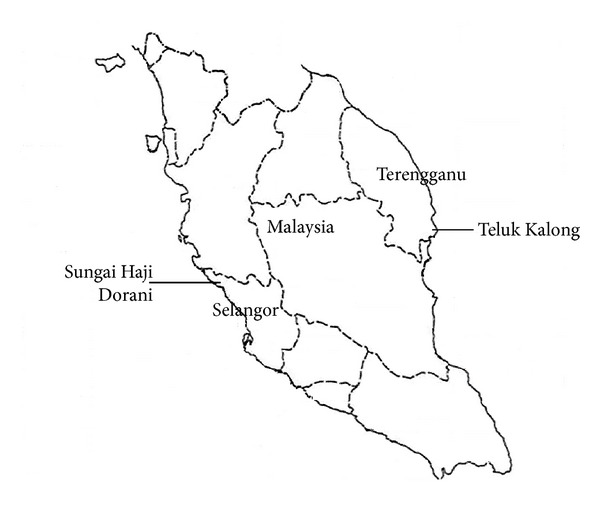
Maps of Peninsular Malaysia showings location of Teluk Kalong and Sungai Haji Dorani.

Teluk Kalong, Terengganu, is located at the east of Peninsular Malaysia. Sandy beaches in Teluk Kalong are popular tourism spots in Malaysia. Wave height along Teluk Kalong is approximately 1.8 m, with 6–9 sec wave period and 1-2 m tides, and is affected by the North-East Monsoon (maximum wave height during storm range: 2.7–4.8 m). Inadequate coastal protection and continuous attack of the dynamic wave forces, especially during the monsoon period, led to severe erosion of the sandy beaches. Besides, the eroded sediment caused the structural instability of the existing precast seawall. Coastal recession also affected the tourism industry in Teluk Kalong. Hence, in 2006, the Public Works Department initiated the remedy project in Teluk Kalong to counteract the coastal retreat. Location of Teluk Kalong is shown in Figure 3.

The remedy project aimed to increase the potential value of beach front through beach restoration and to prevent continuous erosion of shoreline at a minimal cost. Geotextile tubes were utilized in the project due to the ability in coastal protection, speedy installation, simple equipment, and low cost. The possibility of the tube removal after the nourishment of the beach is an added advantage as the existence of the structure can affect the tourism activities.

Geotextile tubes installed in Teluk Kalong were 3.5 m in diameter and placed at 150 m offshore. Total length of the stretches of geotextile tube was 500 m. The geotextile tubes consisted of two layers of synthetic fabrics. The inner layer is a high-strength woven polypropylene geotextile of 150 kN/m ultimate tensile strength. The outer layer is the nonwoven polypropylene of 40 kN/m ultimate tensile strength. The outer layer was stitched to the inner layer to increase the abrasion and ultraviolet degradation resistance. Due to the long term exposure to sunlight, the geotextile used was added with ultraviolet stabiliser such as carbon black to increase the resistance to ultraviolet degradation [23].

These tubes are filled with sand slurry to a height of 2 m and were placed on the scour apron to avoid scouring around the tubes. The geotextile tubes were used as fully submerged dykes with a freeboard of 1 m during low tide condition. The performance of geotextile tubes was not much affected by the currents and the wind factor as they are submerged [24].

**Figure 4:**
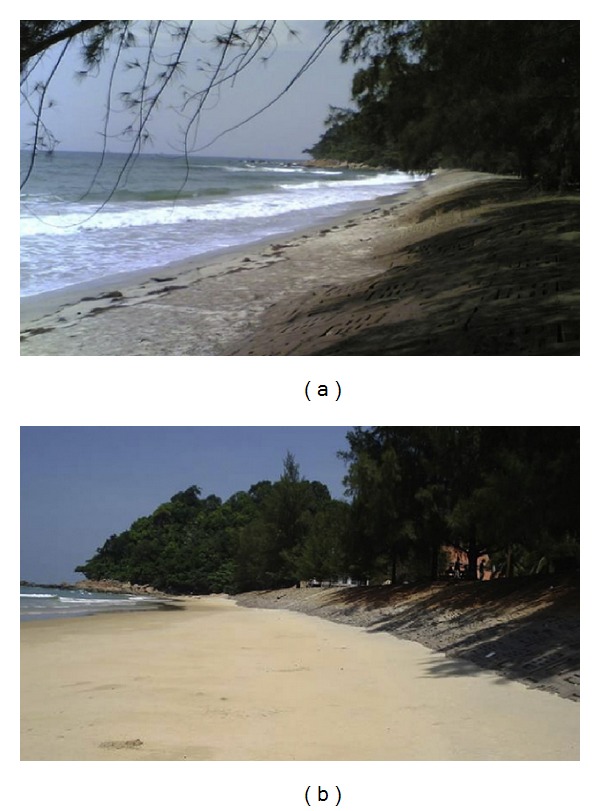
Condition of beach (a) before and (b) after the installation of geotextile tubes.

Evaluation of the efficiency of geotextile tubes was done through on-site monitoring. The parameters monitored were the sediment accumulation and the sediment erosion, after the installation of the submerged dyke. Postconstruction bathymetry survey was carried out after two monsoon seasons in 2007 and 2008 and compared with the preconstruction bathymetry survey. Lee and Douglas [24] reported the average increment of 1.8 m sediment thickness, or estimated accumulation of 87,317 m^3^ of sediments, as the outcome of the project.  Figure 4 shows the conditions of the beach before and after the installation of geotextile tubes.

Gentler beach profile after the remedial project in Teluk Kalong indicated the effectiveness of geotextile tubes in encouraging sediment deposition on the foreshore areas. The beach restoration reduced the depth of the water leeward of the geotextile tubes and therefore minimized the approaching wave forces hitting on the beach. Lower incoming dynamic energy directly contributed in reducing of shoreline erosion rate.

## 3. Application of Geotextile Tubes in Muddy Coasts

Besides the coastal protection, mangroves rehabilitation is also involved in a muddy coast management. Implementations of the geotextile tube in muddy coasts were introduced through the coastal rehabilitation projects in Sungai Haji Dorani, Malaysia, and Chachoengsao, Thailand. Through the cases, the utilization objectives, method, design considerations, and lessons learned were described.

### 3.1. Case I: Muddy Coast of Sungai Haji Dorani, Malaysia

Sungai Haji Dorani, Selangor, is located on the west coast of Peninsular Malaysia, as shown in Figure 3. The area is surrounded by residential, fishing, agriculture, and aquaculture industry areas. Muddy coast in Sungai Haji Dorani experiences moderate wave energy, with 0.5–1.0 m wave height, 6–9 sec wave period, and 2.0–2.5 m tides, and is affected by South-West Monsoon (maximum wave height 2.0-3.0 m). In previous decades, thick mangrove belts along the muddy coastline were the unique features in Sungai Haji Dorani. However, the eroded shoreline led to the death of almost all the mangroves as the roots of mangroves lost grip to the sediment and mangroves toppled over. Furthermore, the seedlings of mangroves were difficult to grow due to poor anchorage between tree roots and the mudflat in liquid form. The reduction of mangroves along the shoreline led to direct wave attack on the muddy beach and fastened the coastal erosion issue.

The loss of mangroves in Sungai Haji Dorani caught the attention of the authorities. Hence, to protect and conserve the mangrove forests, the integrated method through mangrove restoration with the assist of geotextile tubes was introduced. The coastal management project aimed to dissipate approaching wave energy, to encourage sediment accumulation, and to assist the mangrove regeneration. The chief aims of the geotextile tubes were to reduce the wave energy hitting the shoreline and to promote the deposition of sediments for mangrove regeneration [25]. Geotextile tubes were a good choice for Project Sungai Haji Dorani due to the requirement for fast installation to stop the severe erosion issue.

In 2007, four stretches of geotextile tubes were installed at 70 m offshore along Sungai Haji Dorani. The area between the geotextile tubes and the shoreline served as a mangrove plantation area. Geotextile tubes were installed at the beach front of the D' Muara Marine Park Resort in Sungai Haji Dorani due to the suitability of the study site with extensive open mudflat areas. The four high-strength woven geotextile tubes of dimensions 1.8 m × 3.7 m × 50.0 m were filled with sand slurry and placed at a 0.5 m gap between each other. Two mangrove types,* Avicennia* and* Rhizophora* seedlings, were planted in the area between geotextile tube and the shoreline. The two mangrove species have different living preferences.* Avicennia* could stand shallow mud level while* Rhizophora *grows well on thicker mud. Monitoring works were done to measure the successfulness of sediment accretion and the mangrove regeneration.

**Figure 5:**
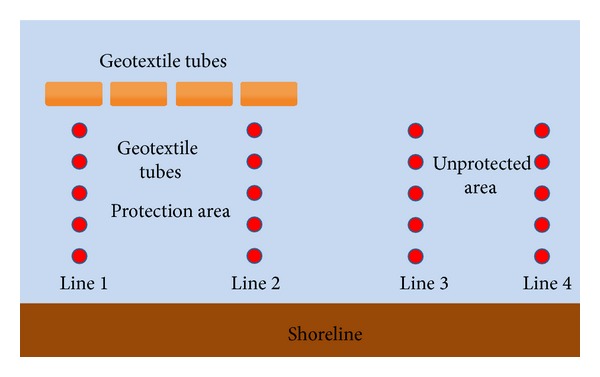
Location of measuring pins behind the geotextile tubes.

Monitoring works were done by observing the measuring pins installed. As shown in Figure 5, there were four monitoring lines. Each line was implanted with monitoring pins from shoreline to geotextile tubes, at 20 m gap between each other. These 0.3 m exposed monitoring pins were plastic pipes implanted along the baseline [26]. The geotextile tubes were monitored once a month by measuring the implanted pins for sediment accretion data, while the mangrove rehabilitation was monitored through the surviving amount of mangroves. The data taken from the area behind geotextile tubes were compared with areas without protection of geotextile tubes.

Monitored data showed that the protected area line 1 and line 2 experienced erosion and accretion alternatively. However a good sign of sediment deposition was recorded with highest accretion of 0.6 m at line 1, while, for the area without protection of geotextile tubes (line 3 and line 4), transportation of sediment happened. When line 3 experienced erosion of 0.4 m, line 4 had sediment accretion of 0.4 m. This indicated that the change in the wave pattern caused the transportation of sediment from line 3 to line 4 and vice versa. Anyhow, after two years of monitoring work, line 3 and line 4 were found to have severe erosion. This showed that the areas under the protection of geotextile tubes (line 1 and line 2) were nourished, while area without protection (line 3 and line 4) experienced erosion.

Besides sediment transportation, mangroves were monitored. Amount of mangroves survived and death was recorded. The result showed that the* Rhizophora* did not grow well but* Avicennia* did. Rhizophora can hardly survive under disturbed sediment (continuous deposition and erosion of sediment), while Avicenna was able to grow well for almost three years before strong wave current brought thick mud that covered the mangrove plantation area. This caused the destruction of many healthy Avicennia.

Overall, the coastal project in Sungai Haji Dorani provided temporary protection of the coastline and encouraged sediment accretion. Sediment accumulation stabilized the shoreline and provides a suitable area of mangroves planting [27]. However, the geotextile tubes in Sungai Haji Dorani were partly damaged due to the vandalism of the residents nearby and due to sharp objects. Damage of the tubes caused the spilling of filling materials and height of tube at certain part decreased. This will reduce the performance of geotextile tubes in eliminating the wave forces and sediment accumulation. Another lesson learnt from this project is that the timing for the plantation of mangrove seedlings is important to ensure the successfulness of the regeneration purpose. Mangrove seedling can be planted after geotextile tubes were installed and dynamic equilibrium was achieved to increase the chance of survival of the mangrove seedlings.

### 3.2. Case II: Muddy Coast of Chachoengsao, Thailand

**Figure 6:**
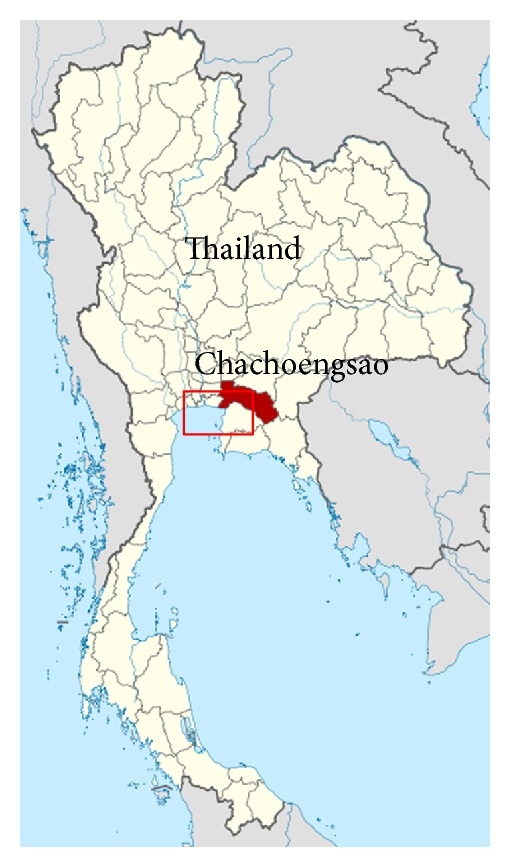
Maps of Thailand showing eroded area at Chachoengsao's coast.

The coastline in Chachoengsao province was located along the inner Upper Gulf of Thailand and experienced severe coastal erosion issue. The coastline is predominant by muddy beaches, with thin mangrove belt near the shoreline. Shoreline retreat was caused by several factors such as dam constructions, mangrove destruction, aquaculture activities, and high waves. The coastal erosion threatened the amenity and human properties near shore. The red box in Figure 6 shows the location of geotextile tubes installed at Chachoengsao's coast.

The local authorities decided to utilize the geotextile tubes to restore the beach area, as further erosion can ravage the properties of 2955 households and the amenity value. There were several considerations for the design of protection structure along Chachoengsao's muddy coast which included the soft and weak foundation and no access road for material transportation. Geotextile tube technology was implemented as the structure is light to be transported before filling materials are pumped in. Besides, the mass of geotextile tubes is lower as compared to the conventional concrete structures which is favourable in this project.

Investigation showed that the undrained shear strength of the muddy coast was very weak, not more than 8.5 kN/m^2^. Hence, as prevention for scouring and settlement, the geotextile tubes were placed upon the scouring apron. Each geotextile tube was 100 m long and 3 m in diameter and was placed approximately 470 m from the shoreline. The geotextile tubes were designed to have a +1.6 m crest elevation from mean sea level.

Saengsupavanich [28] reported the effective coastal protection for four years after the installation of geotextile tubes in Chachoengsao's muddy coast. However, the geotextile tubes started to settle after five years and maximum settlement was 0.6 m. Thus, the incident waves, especially during the storm surges, overtopped the geotextile tubes and continuously eroded the shoreline. The local authorities then decided to cover the geotextile tubes with layers of rocks to increase the structure's height, which cost USD 500 per meter. Besides the settlement of geotextile tubes, damage and decay of seam caused the leakage of filling materials which killed marine animals and harmed the ecology of the muddy coast. Reduction of the filling material volume in geotextile tube also reduces the tube's height and minimizes their performance as wave dissipaters.

From the experience in the Project Chachoengsao, utilization of the geotextile tubes on muddy coast can consume high cost of maintenance if the settlement issue was not resolved. Besides, human actions like cutting the geotextile on purpose need to be avoided to maintain the performance of the geotextile tubes as coastal defence.

## 4. Discussions

The application of geotextile tubes in sandy and muddy coasts was introduced through case studies. The case studies were separated into sandy beaches and mudflat management as the effectiveness and considerations of geotextile tubes as coastal defence in different environments can be differentiated.

### 4.1. Differences in Sandy Coast and Muddy Coast

Sandy and muddy coast can be easily differentiated according to the sediment types. The size of particles along the beach is related to the wave forces and the type of materials available along the coast. Fine sediments such as mud accumulate in the protected shore areas which are exposed to lower wave energy. Adversely, coarser sediments such as sand deposit along the coast that is exposed to higher dynamic forces. For instance, Teluk Kalong's sandy beaches experienced higher wave energy (1.8 m weight height) in comparison to mudflat in Sungai Haji Dorani (0.5–1.0 m wave height). The designs of geotextile tube for sandy beach and mudflat are definitely different in terms of the strength and height of the tube. For example, geotextile tube in sandy beach requires higher strength and design height to withstand the higher wave energy.

Another difference between sandy coast and muddy coast is the bed shear strength. Sandy coast has higher bed shear strength as compared to muddy coast. Saengsupavanich [28] reported 14 m loose mud (SPT-Value = 0) from the soil surface in Chachoengsao's muddy coast, with the bed shear strength less than 8.5 kN/m^2^. Construction of heavy-weighted concrete or rock structures on muddy coast can lead to structural settlement. Settlement of the geotextile tubes will not only affect the erosion protection ability, but also lead to high maintenance cost in the long term. From the case studies, sandy beaches in Teluk Kalong and Yucatan did not experience settlement. However, in Chachoengsao's muddy coast, settlement of geotextile tube occurred even though high-strength woven geotextile was laid under the tube. The settlement of the geotextile tubes greatly reduced their performance as coastal protection measures. Hence, to overcome the settlement issue, successful experience from Hashim et al. [29] where concrete breakwater was laid upon bamboo mat to minimize the structure settlement in muddy coast can be adapted. Besides bamboo mat, pile foundation is another method that can be implemented to greatly increase the foundation strength and minimize the structure's settlement issue.

Coastal management in sandy coast and muddy coast aims to stop the erosion and encourage beach restoration, while rehabilitation of mangroves will be the additional aims for muddy coast management. Mangroves are unique plants that can be found in muddy coasts of many tropical countries. Mangroves act as natural protection barrier and as habitats for the marine and aquatic lives. Destruction of mangroves due to the overexploitation and eroded muddy coast allowed the waves to directly hit on the soft muddy shore and accelerate the sediment depletion. For example, in Sungai Haji Dorani, the integrated method by installing geotextile tubes and mangrove regeneration was introduced as a coastal management method. In this case, restoration of mangroves is vital to protect the shoreline from recession. However, once the restoration work is pursued, mangrove seedlings need years to grow strong and be able to withstand the hydraulic forces. Hence, assistance from coastal structures is necessary to dissipate the wave energy to ensure the survival of mangroves.

### 4.2. Beach Replenishment

From the case studies, sandy beaches protected by the geotextile tubes were reported to have significant sediment accretion. Sediment accretion ranging from 0.45 m to 0.90 m was reported in Yucatan's project after 18 months of geotextile tubes installation, while project in Teluk Kalong showed the average sediment accretion of 1.8 m, or accumulation of 87,317 m^3^ of sediment, after two years of geotextile tubes installation.

Muddy coast management using geotextile tubes in Sungai Haji Dorani and Chachoengsao overall provides effective erosion prevention with several issues. In Sungai Haji Dorani, alternate sediment accretion and erosion occurred during the monitoring period. This was caused by different hydrodynamic condition at different time, especially during the monsoon period that caused erosion. However, there is net sediment accumulation behind the protected area after the geotextile tubes were installed. Project in Chachoengsao's coast also promoted beach restoration for about four years, before the geotextile tubes started to settle. Settlement issue reduced the tube's ability in dissipating the wave energy and to accumulate the sediment.

Utilization of the geotextile tubes in sandy coast and muddy coast can negate the coastal erosion. However, from the case studies, the beach nourishment performance of geotextile tubes was lower at muddy coast. Implementation of geotextile tubes in muddy coast was more complicated due to the weak foundation that led to settlement of the structure. Settled geotextile tubes had lower ability in dissipating the turbulences and accumulate the sediments around the structures. Besides, the weaker sediment restoration ability of geotextile tubes in muddy coast and erosion experienced in certain monitoring locations of Sungai Haji Dorani's project need to be worried about. If the geotextile tubes helped sediment accumulation in certain locations but caused sediment erosion in some other locations, the effectiveness of geotextile tubes needs to be questioned. Design considerations such as the structure's height, placement and the locations of the geotextile tubes, and the littoral drift direction need to be carefully considered.

### 4.3. Mangrove Rehabilitation

Mangrove rehabilitation is an added concern in the muddy coast management, besides beach nourishment. Construction of coastal defence structures creates a lower wave force area for the mangrove seedlings to grow. Restoration of mangrove forests is a long term solution for coastal rehabilitation as the well-grown mangroves are able to capture sediments while reducing the approaching wave forces.

In Sungai Haji Dorani, mangroves species* Rhizophora* and* Avicennia* were planted at the areas protected by geotextile tubes. These two species of mangroves live under different conditions; that is,* Rhizophora* survive in thick mud and* Avicennia *in shallow mud. The* Rhizophora* seedlings did not survive during the project as the sediment accreted and eroded alternatively. Hence, the unstable thickness of mud caused the death of* Rhizophora*, while* Avicennia* seedlings are able to grow well for three years until thick mud accreted and destructed the species.

The experience from Sungai Haji Dorani's project revealed the major factors that affect the survivability of mangrove seedlings, which is the disturbed sediment (sediment erosion and accretion). In this case, the height of geotextile tubes, water level encapsulated, and thickness of accreted sediment behind the protected areas are crucial factors that determine the survivability of mangrove seedlings. Therefore, the design of geotextile tubes must be performed accordingly to produce the appropriate environment for the selected mangrove species. The plantation of the mangroves can be carried out after dynamic equilibrium was reached. Rate of sediment accretion and erosion around the geotextile tubes will be lower when dynamic equilibrium was achieved. Hence, the mangrove seedlings will have a higher chance of survival due to the minimal sediment disturbance.

### 4.4. Construction Cost

Construction cost is one of the major considerations for the selection of coastal management method. Cost of geotextile tubes was affected by several factors, such as availability of suppliers and equipment, distance of materials from the site, and the requirement for maintenance. According to Russell and Michaels [30], the cost for the geotextile tubes breakwater in Malaysia was approximately USD 700,000 per kilometre, which was higher than other tropical countries like Vietnam, USD 300,000 per kilometre. The installation cost of a geotextile tube was higher in Malaysia as compared to other countries like the United States, Australia, and Germany due to the lack of suppliers and equipment.

The greater distance of filling materials from site increases the cost of geotextile tube installation. As reported by Howard et al. [31], the filling materials for geotextile tube in Tanjung Piai were purchased with freight distance and hence the cost was less economically favourable. Besides, issues like structure settlement or failure can lead to high maintenance cost. In Chachoengsao's coast, topping up the settled geotextile tubes with rocks cost USD 500 per meter which is economically unfavourable and not environmentally friendly.

Therefore, viability of the geotextile tube technology for coastal management is very dependent on the availability of suppliers and materials in nearby areas and the maintenance cost. Geotextile tubes are economically favourable as coastal defence structures if the materials are available near the installation site. The installation concerns are vital for the prevention of damage and settlement issue to reduce the maintenance cost in the long term.

### 4.5. Advantages and Disadvantages of the Geotextile Tubes

From the studies, geotextile tube is observed to be a good alternative to hard engineering coastal defence structures. Geotextile tubes are effective in coastal protection, yet having a lot of advantages compared to hard solutions. Installation of geotextile tubes enables the nourishment of the beach with minimal time because only simple equipment and procedures are involved. Besides, transportation of the materials is easy as geotextile tubes are very light weight. The geotextile tubes are very flexible structures as they can be removed any time when no longer needed [32]. Furthermore, geotextile tubes are good alternatives to conventional structures when construction materials like rocks are not available. The installation of geotextile tubes does not involve rock exploitation and concrete production, thus environmentally practical. Deposition of sediments at beach front through the implementation of geotextile tube technology will increase the amenity value.

Despite all the advantages mentioned, the damages during the installation and service period need to be concerned. Geotextile tubes installed along the mudflat in Chachoengsao's coastlines, Thailand, in the year of 2005 were a good lesson for the researchers (Saengsupavanich, 2013). The geotextile tubes installed experienced 0.6 m settlement after five years and were damaged. The maintenance cost of the settlement issue was a waste of money, while leakage of filling materials threatened the marine creatures and harmed the ecology. Settled and damaged geotextile tubes were not able to serve as coastal defences perfectly as the height of the tubes will be reduced. Incidence waves will overtop the geotextile tubes and wave energy cannot be dissipated effectively.

Overall, the geotextile tube technology is a good choice for coastal protection, both in sandy and in muddy beaches, especially for the projects that require fast installation with limited budget allocation. Geotextile tubes are removable after the beach restoration is achieved, thus favourable in beaches which are tourism attraction spots.

After the studies, there are several recommendations to refine the applications of geotextile tubes for sandy and muddy coasts management. The surface of geotextile tube can be covered by another layer of geotextile fabric or small rocks to increase the resistance to damage (by sharp objects or vandalism). Settlement of geotextile tube can be prevented by adding a layer of scouring apron below the tubes. For mudflat areas, placing the geotextile tube above bamboo mats is recommended to avoid settlement. Close liaison between scientists and engineers is very important to ensure the geotextile tube installation is able to assist the regeneration of mangrove species.

## 5. Conclusion

In this paper, case studies regarding the application of geotextile tubes in both sandy and muddy coasts were carried out. The coastal management experiences from Mexico, Malaysia, and Thailand were elaborated and discussed further for the application of geotextile tubes.

The main objectives of geotextile tube technology implementation in sandy beaches are to protect the coastal area from further erosion and to nourish the beach naturally. However in muddy coasts, mangrove regeneration is an extra concern in coastal management projects. Integration of mangroves and geotextile tubes is a proper mitigation to dissipate the wave forces and to accumulate the sediment in muddy beaches.

Sandy beach and muddy beach have different geological settings. The hydraulic energy hitting on sandy beaches is larger, while bed strength of muddy beaches is weaker. Therefore, different considerations are required for the design of geotextile tube in these two coast types. Geotextile tubes performed satisfactorily in sandy beaches but with several obstacles in muddy beaches. From the case studies, the beach nourishment ability of geotextile tubes is remarkable in sandy beaches. However in muddy beach, the geotextile tubes were able to minimize the shoreline erosion with issues. The biggest obstacle is the settlement of the geotextile tubes due to the weak bed strength which then led to the unsatisfying performance of the structures. Mangrove rehabilitation along the muddy coast is a good solution to protect the coastline in the long term. However, the successfulness of the regeneration of mangroves is depending on the assistance of the geotextile tubes. Hence, design considerations such as placement of the tubes and when to plant the mangroves are very crucial to ensure the successfulness of coastal and mangrove rehabilitation.

Overall, advantages of the geotextile tubes such as fast execution, low cost, light weight, simple equipment requirement, and effectiveness in coastal protection made them good alternative for hard engineering structures. However, the lower resistance of geotextile to damage needs to be overcome to ensure the longer service period of the geotextile tubes. Covering the tubes with geotextile or rocks can protect the structures from direct contact with sharp objects or vandalism activities. Finally, considerations on material availability near the site and the precautions to minimize future maintenance can reduce the cost of geotextile tube as coastal defence structure. Therefore, preservation and rehabilitation of beach front by geotextile tubes are environmentally and economically viable, in both sandy and muddy coasts.
